# Short-term co-ingestion of creatine and sodium bicarbonate improves anaerobic performance in trained taekwondo athletes

**DOI:** 10.1186/s12970-021-00407-7

**Published:** 2021-01-21

**Authors:** Amir Sarshin, Vahid Fallahi, Scott C. Forbes, Alireza Rahimi, Majid S. Koozehchian, Darren G. Candow, Mojtaba Kaviani, Seyed Nemat khalifeh, Vahid Abdollahi, Alireza Naderi

**Affiliations:** 1 Faculty of Physical Education and Sport Sciences, Department of Exercise Physiology, Karaj Branch, Islamic Azad University, Karaj, Iran; 2 Faculty of Education, Department of Physical Education, Brandon University, Brandon, MB R7A6A9 Canada; 3 Department of Kinesiology, Jacksonville State University, Jacksonville, AL 36265 USA; 4 Faculty of Kinesiology and Health Studies, University of Regina, Regina, SK S4S0A2 Canada; 5 School of Nutrition and Dietetics, Acadia University, Wolfville, Nova Scotia Canada; 6 Taekwondo National team, Taekwondo federation, Tehran, Iran; 7 Department of Sport Physiology, Boroujerd Branch, Islamic Azad University, Boroujerd, Iran

**Keywords:** Taekwondo, Performance, Creatine, Sodium bicarbonate, Taekwondo anaerobic intermittent kick test

## Abstract

**Background:**

Creatine (CR) and sodium bicarbonate (SB) alone improve anaerobic performance. However, the ergogenic effects of CR and SB co-ingestion on taekwondo anaerobic performance remains unknown.

**Methods:**

Forty trained taekwondo athletes (21 ± 1 y.; 180.5 ± 7.3 cm; 72.7 ± 8.6 kg) were randomized to: (i) CR and SB (CR + SB; 20 g of CR+ 0.5 g·kg^− 1^·d^− 1^ of SB), (ii) CR, (iii) SB, (iv) placebo (PLA), or (v) control (CON) for 5 days. Before and after supplementation, participants completed 3 bouts of a Taekwondo Anaerobic Intermittent Kick Test (TAIKT) to determine changes in peak power (PP), mean power (MP), and fatigue index (FI). Blood lactate (BL) was measured before, immediately following, and 3 min post-TAIKT.

**Results:**

PP and MP increased over time (*P <* 0.05) following CR + SB, CR, and SB ingestion, with no changes in the PLA or CON groups. There was a greater increase over time in MP following CR + SB (Absolute Δ: 1.15 ± 0.28 W∙kg^67^) compared to CR (Absolute Δ: 0.43 ± 0.33 W∙kg^67^; *P <* 0.001) and SB (Absolute Δ: 0.73 ± 0.24 W∙kg^67^; *P* = 0.03). There were no significant time and condition effect for FI (*P* > 0.05). BL increased following exercise across all groups; however, CR + SB and SB post-exercise BL was lower compared to CR, PLA, and CON (*P* < 0.05).

**Conclusion:**

Short-term CR and SB alone enhance TAIKT performance in trained taekwondo athletes. Co-ingestion of CR and SB augments MP compared to CR and SB alone, with similar PP improvements.

## Introduction

Taekwondo is a combat sport that has been in the Olympic games since 1988 and is growing in popularity. Taekwondo involves short bursts of high-intensity activity (~ 1–5 s) interspersed with low-intensity movements (1:2–1:7 ratio) [1]. A taekwondo match is comprised of three two-minute rounds interspersed with one-minute of passive recovery. Due to the length, intensity, and intermittent nature of a taekwondo match, athletes require well-developed non-oxidative (phosphocreatine and glycolytic) and oxidative energy systems [2–4]. Throughout a match, the relative contribution from the anaerobic glycolytic system increases due to the short recovery between rounds [3, 4]. In particular, hydrogen ions (H^+^) may accumulate during a match and potentially impair performance [5]. Specifically, H^+^ impair exercise performance through inhibition of key glycolytic enzymes (i.e., phosphorylase and phosphofructokinase), impaired calcium handling, and reduced myosin ATPase activity [6].

Dietary strategies including supplements known to alter either extracellular or intracellular buffering capacity or alter the contribution from the glycolytic energy system may enhance taekwondo performance [7]. Sodium bicarbonate (SB) supplementation improves extracellular buffering capacity and enhances high-intensity exercise [8], including combat sports performance [9–12]. Lopes-Silva (2018) demonstrated that acute SB supplementation (300 mg∙kg^− 1^) increased glycolytic metabolism and enhanced taekwondo performance [3].

Another potential dietary strategy is creatine (CR) supplementation. CR is an organic compound naturally produced in the body from reactions involving arginine, glycine, and methionine in the kidneys and liver [13]. CR supplementation increases phosphocreatine stores within the muscle by ~ 20% [13]. The breakdown of phosphocreatine via creatine kinase supports ATP rephosphorylation during high intensity or explosive activities [6]. The phosphocreatine energy system contributes to ~ 26–30% of the energy requirements during action phases in taekwondo [14]. Mechanistically, CR can act through several mechanisms including ATP rephosphorylation, and an intracellular buffer [6, 15].

In theory, co-ingestion of SB and CR may provide additional ergogenic effects since they act through different mechanisms. In a crossover design, Barber et al. (2013) supplemented trained males (*n*= 13) with CR (20 g) and SB (0.5 g/kg) for only 2 days (with a relatively short 3-week washout) compared to either CR or PLA alone. Mean and peak power was higher during repeated sprints following co-ingestion compared to CR or placebo (PLA) alone [16]. Six days of CR Co-ingestion (20 g∙day^− 1^) and an acute dose of SB improved swimming performance by 1.5% in the second repeat of a 2 × 100-m freestyle swimming test compared to PLA [17]. In contrast, Griffen et al. (2015) reported no additional effect of CR and SB when compared to CR and SB alone on indices of mechanical power in well-trained males (*n* = 9) performing repeated Wingate tests using a cross over design with a 7-day washout between conditions [18]. A recent meta-analysis reported that chronic (500 mg∙kg^− 1^ for 5 d) but not an acute bolus of SB improved peak and mean power during anaerobic performance [19]. To date, studies have provided mixed results that may be associated with methodological limitations, such as short washout periods (≤ 3 wks), acute SB dosing, and lack of comparison groups. Therefore, the purpose of this study was to examine the effects of CR monohydrate and SB co-ingestion on mean power (MP), peak power (PP), fatigue index (FI), rating of perceived exertion (RPE), and blood lactate (BL) during a sport-specific anaerobic intermittent kick test (TAIKT) in trained taekwondo athletes compared to CR, SB, PLA, and control (CON). We hypothesized that SB and CR co-ingestion would increase MP and PP compared to CR, SB, PLA and CON. It was also hypothesized that CR and SB ingestion alone would enhance anaerobic performance compared to PLA and CON.

## Methods

### Participants

Fifty-five male professional taekwondo athletes actively competing in the national taekwondo league volunteered to participate in this study. Participants had an at least of 6 y immediate prior history of playing taekwondo, and they had ≥2 yrs experience of competing in the Iran's taekwondo league. The general characteristics of participants are presented in Table 1. To ensure participants were “trained”, the skill level of participants was verified by experienced Iranian taekwondo national team coaches. Participants were excluded if they were taking medications that could influence muscle performance (i.e., corticosteroids), had ingested CR monohydrate or any Cr containing dietary supplements within 12 weeks prior to the begining of the study, if they were vegetarian, had an orthopedic injury, or if they had pre-existing kidney or liver impairments. A health history questionnaire (HHQ) and a physical activity readiness questionnaire (PAR-Q) were administered to ensure that participants were safe to perform high-intensity physical activity [20]. After initial screening, 40 taekwondo athletes met the inclusion criteria and were invited to participate in the study. All participants were in the general preparation phase of the yearly training program. Participants were engaged in six training sessions per week including four taekwondo specific training sessions and two specific strength and conditioning sessions. Participants were instructed not to change their habitual diet or physical activity beyond their normal daily routines. Participants also were asked to perform a light recovery training session 48 h before each test and to avoid vigorous exercise. The research ethics board approached the study at Islamic Azad University, Karaj Branch, Karaj, Iran (IR.IAU.K.REC.1399.04.09). Participants were informed of the risks, benefits, and purpose of the study before the written consent was obtained.

**Table 1:** General characteristics of Participants

	CR + SB	CR	SB	PLA	CON
**Age (years)**	22.9 ± 1.8	21.4 ± 1.1	23.1 ± 2.4	22.6 ± 1.7	22.4 ± 1.7
**Body Mass (kg)**	73.6 ± 8.5	72.0 ± 11.0	71.5 ± 10.9	72.2 ± 7.2	74.6 ± 6.4
**Height (cm)**	182.5 ± 10.5	181.3 ± 5.1	179.3 ± 6.5	180.1 ± 8.4	179.8 ± 6.3
**Training experience (yrs)**	9.5 ± 1.2	8.6 ± 2.6	9.1 ± 2.2	8.3 ± 1.5	8.5 ± 1.5

### Experimental approach to the problem

The cuurent study was conducted in randomized, repeated measures placebo-controlled manner. Participants were required to attend the laboratory on four occasions. Prior to each testing session, participants were asked to wear light and comfortable clothing, refrain from vigorous exercise for 48 h, and avoid consuming caffeine and alcohol for 24 h. During the first visit, height and body mass were assessed by an electronic stadiometer (SECA 217; SECA Ltd., Hamburg, Germany) and a calibrated digital scale (Seca 770; Seca, Hamburg, Germany), respectively. In the second visit, participants were familiarized with the Taekwondo Anaerobic Intermittent Kick Test (TAIKT) [4]. Approximately 72 h later, participants returned to the laboratory for pre-testing and completed three rounds of the TAIKT interspersed with 1-min passive recovery to simulate an official taekwondo match. PP, MP, and FI were assessed during each round [4]. The well-being Hooper index questionnaire [21] was used before each TAIKT to monitor subjective feelings of recovery and fatigue. Before, immediately and 3-min after the TAIKT, BL was determined photometrically using a portable analyzer (Lactate Scout+ analyzer, SensLab GmbH, Germany). Furthermore, ratings of perceived exertion (RPE) were determined after each bout [22]. After baseline (pre-test) testing participants were matched based on their successful kick performance, fitness and technical level (assessed by a professional coach not involved in the present study) prior to randomization into one of five groups: [1] creatine monohydrate and sodium bicarbonate (CR + SB; *n* = 8), [2] creatine monohydrate and placebo (CR; *n* = 8), [3] sodium bicarbonate and placebo (SB; *n* = 8), [4] placebo and placebo (maltodextrin) (PLA; *n* = 8), and [5] control (CON; *n* = 8). Participants ingested the supplements for five consecutive days. To minimize the potential of training volume, coaches were informed of the purpose of the study and were asked to perform maintenance training volume throughout the study.

### Supplementation protocol

The supplements were isovolumetric and similar in color, taste and appearance. Participants were randomized into the following groups: co-ingestion of CR (20 g∙day^− 1^, bulkpowders.de, Germany) and SB (500 mg∙kg^− 1^, AGC Industries Co., China), CR (20 g∙day^− 1^ and 500 mg∙kg^− 1^ maltodextrin [GLUCIDEX® 12 Roquette Industries Co., France], SB (20 g∙day^− 1^ maltodextrin and 500 mg∙kg^− 1^ SB), PLA (20 g∙day^− 1^ maltodextrin and 500 mg∙kg^− 1^ maltodextrin), or CON (receiving no supplementation) [18, 19], for 5 days before returning to the laboratory for post-testing (repeat of pre-testing). Maltodextrin was added to all conditions to ensure that participants received isovolumetric supplements [16, 18] and to facilitate CR uptake [23, 24]. These dosing strategies for CR and SB have been shown to elevate muscle CR [25] and blood bicarbonate (HCO_3−_) [12], respectively. To minimize the potential gastrointestinal side effects of SB, a split-dose strategy was employed [26]. The total daily SB and CR doses was divided into 4 equal portions to be consumed throughout the day at 9 a.m., 12 p.m., 5 p.m., and 9 p.m. [16, 18]. Participants were instructed to mix each supplement sachet with 330 mL of water diluted with 40 mL of orange cordial [18]. A gastrointestinal questionnaire was used to evaluate the symptoms of discomfort [27] by providing a score ranging from 0 to 9, where 0 was ‘no problem at all,’ and 9 was ‘the worst it has ever been’. The symptoms were considered severe when a score ≥ 5 [27]. Participants recorded their diet prior to pre-testing were instructed to duplicate the diet 24 h before post-testing [9, 16]. Furthermore, participants consumed a standardized snack including 1.5 g of carbohydrate per kg of body mass, 150 min before each TAIKT.

### Taekwondo anaerobic intermittent kick test (TAIKT)

The TAIKT is a valid and reliable sport-specific anaerobic test to determine peak and mean power output and fatigue index in taekwondo athletes [4]. TAIKT has excellent reliability (ICC > 0.90) with the Running-based Anaerobic Sprint Test and Wingate test [4, 28]. According to an official taekwondo match, participants completed 3 bouts interspersed with 1-min of passive recovery [1]. Prior to the TAIKT, a 15 min standardized warm-up was performed and consisted of 2 sets of 20 alternating front kicks (Ap-Tchagui) and 3 sets of 20 alternating Bandal-Tchagui on a Taekwondo Pao at a moderate rhythm [4], followed by 5 min of passive recovery. All participants performed the testing in official taekwondo official protectors [4]. As previously described [4] each TAIKT bout consisted of 6-sets of 5-s maximal stationary roundhouse kicks (Bandal-Tchagui) on an electronic body protector placed around a hanging punching bag. Each 5-s set was separated by 10 s of active recovery (bouncing movements). The hanging punching bag was placed at the level of the participant’s trunk at a height (y) relative to the mat, which was recorded and replicated for post-testing. Participants also determined an optimal horizontal distance (x) from the punching bag based on performing 10 roundhouse kicks from a ready stance [4]. The horizontal distance was marked with adhesive tape on the mat. During the execution of the kicks participants were required to start and finish from the “X” [4]. The TAIKT began and ended with a sound signal. The number of adequately executed kicks was automatically displayed on the computer screen. The horizontal distance (x) and trunk height (y) were used to calculate the projection distance of the foot (d) on the body protector using Pythagorean theorem (d = √x^2^ + y^2^) [4]. Lower limb mass (LLM) (eq. 1) [29], number of kicks in each 5-s set, and the total number of kicks in each bout were used to calculate the absolute power (eq. 2) [4]. Allometric scaling (W∙kg^-0.67^) [30] was used to determine relative PP (eq. 3), MP, and FI in each separate bout, as well as total peak power (all three rounds combined) [4]. In each round, PP was the highest power output achieved during an individual set, MP was the average power output across all six sets, and FI was the difference between PP, and minimum power output divided by the total test duration (6-sets of 5 s = 30 s).

1 $$ \mathrm{LLM}=\left(\left[\mathrm{thigh}+\mathrm{lower}\ \mathrm{leg}+\mathrm{foot}\ \mathrm{percentages}\right]\times \mathrm{body}\ \mathrm{mass}\ \left[\mathrm{kg}\right]\right)/100 $$

2 $$ \left(\mathrm{Absolute}\ \mathrm{Power}\ \left[\mathrm{W}\right]\right)=\mathrm{LLM}\times {\left(\mathrm{d}\times \mathrm{number}\ \mathrm{of}\ \mathrm{kicks}\ \mathrm{in}\ \mathrm{a}\ \mathrm{set}\right)}^2/{\left(5\ \sec \right)}^3 $$

3 $$ \left(\mathrm{Relative}\ \mathrm{Power}\ \left[\mathrm{W}\ {\mathrm{kg}}^{-0.67}\right]\right)=\mathrm{Abs}\ \mathrm{Power}\ \left(\mathrm{W}\right)/{\mathrm{kg}}^{0.67} $$

### Blood lactate measurement

Blood samples were collected from participants’ earlobe before warm-up, immediately following, and 3 min post-TAIKT. Blood lactate concentrations were determined photometrically by a portable analyzer according to the manufacturer instructions (Lactate Scout^+^ analyzer, SensLab GmbH, Germany).

### Statistical analysis

All results are reported as mean and standard deviations. The normality of the data was checked by using the Kolmogorov-Smirnov test. A three factorial ANOVA with repeated measures (bout 1 vs. bout 2 vs. bout 3) x group (CR + SB vs. CR vs. SB vs. PLA vs. CON) x time (baseline vs. post-testing) was used to determine differences in variables (PP, MP, FI, BL, and RPE). Total scores (combining all three bouts) for each variable were analyzed with a group x time repeated measures ANOVA. If significant interactions were detected, Tukey’s post hoc comparisons were applied to identify where differences occurred. A one-way ANOVA was used to determine differences between groups for absolute change (post value minus pre-value) and relative change (post vale – pre-value/pre-value*100) for total scores. Significance was set a priori at an alpha level of *p* ≤ 0.05. The magnitude of the difference between significant means was determined by eta squared (${\eta}_P^2$). This is a measure of the effect size and therefore of the proportion of the total variance that can be explained by the effects of the treatment. A η^2^ value of 0.15 represents large differences, 0.06 represents medium differences, and 0.01 represents small differences [31]. Statistical analyses were performed using Statistica 13 (StatSoft, Tulsa, Okla.).

## Results

Participants' characteristics are shown in Table 1. There were no differences between groups for any variable at baseline (*P* > 0.05). There was a significant group x time interaction for body mass (*P* < 0.001, ${\upeta}_{\mathrm{P}}^2$ = 0.735). Post hoc analyses revealed that only the CR conditions increased body mass over time (CR + SB: Pre = 73.6 ± 8.4 kg, Post = 74.6 ± 8.3 kg; CR: Pre = 72.0 ± 11.0 kg, Post = 72.9 ± 11.2 kg, *P* < 0.001), with no other changes (SB: Pre = 71.5 ± 10.9 kg, Post = 71.5 ± 10.9 kg; PLA: Pre = 72.2 ± 7.2 kg, Post = 72.3 ± 7.2 kg, CON: Pre = 74.6 ± 6.4 kg, Post = 74.5 ± 6.5 kg, *P* > 0.05). FI did not change between groups over time over time between groups (*P* > 0.05). There was no reported gastrointestinal discomfort and the Hooper score was unaltered (*P* > 0.05).

### TAIKT

#### Peak power

**Fig. 1:**
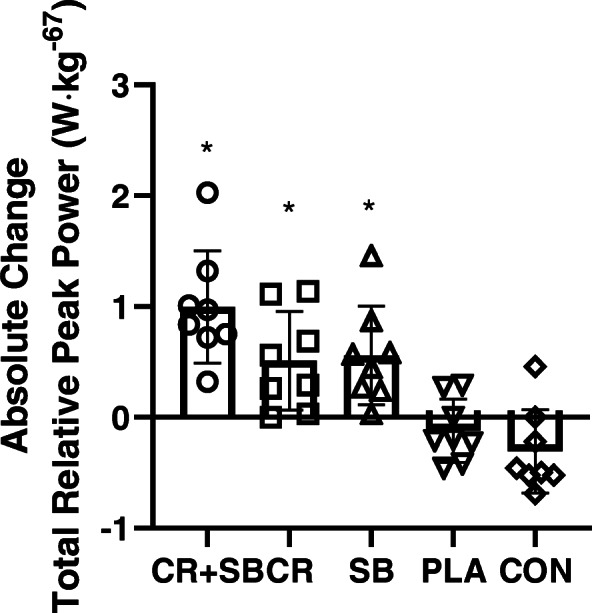
Mean and SD for absolute total relative peak power change from baseline to post testing. Each dot represents an individual participant. * = significantly different than PLA and CON

There was a significant bout x group x time interaction for peak power (*P* < 0.001, ${\eta}_P^2$ = 0.366), as shown in Table 2. Post hoc analyses revealed a significant increase over time in bouts 1 and 2 following CR + SB (*P* < 0.001), with no other changes (*P* > 0.05). There was a significant group x time interaction for total (all three bouts combined) peak power (*P* < 0.001, ${\eta}_P^2$ = 0.600). Post hoc analysis revealed a significant increase over time for CR + SB (*P* < 0.001), CR (*P* = 0.004), and SB (*P* = 0.02), with no changes for PLA and CON. Absolute and percent change scores from baseline to post testing between groups was significant for total peak power (Absolute: *P* < 0.001, ${\eta}_P^2$ = 0.600; Percent: *P* < 0.001, ${\eta}_P^2$ = 0.600). Post hoc analysis revealed greater (*P* < 0.05) change scores for CR + SB (Percent Δ: 24.8 ± 10.9%), CR (Percent Δ: 13.8 ± 12.7%), and SB (Percent Δ: 14.9 ± 11.2%) compared to PLA (Percent Δ: − 3.5 ± 7.2%) and CON (Percent Δ: − 6.9 ± 9.4%), with no differences between supplemental groups (i.e., CR + SB, CR, SB). The absolute changes are shown in Fig. 1.

**Table 2:** Mean and SD for peak power, mean power, fatigue index, and successful kicks in each round at baseline and post-testing for each group

		Bout 1		Bout 2		Bout 3	
		Baseline	Post-Test	Baseline	Post-Test	Baseline	Post-Test
**Peak Power (W∙kg**^**-0.67**^**)**	**CR + SB**	1.30 ± 0.15	1.74 ± 0.23^a^	1.33 ± 0.11	1.68 ± 0.34^a^	1.37 ± 0.22	1.57 ± 0.25
	**CR**	1.36 ± 0.14	1.52 + 0.18	1.27 ± 0.19	1.47 ± 0.23	1.27 ± 0.14	1.42 ± 0.10
	**SB**	1.28 ± 0.16	1.47 ± 0.22	1.28 ± 0.18	1.47 ± 0.26	1.27 ± 0.25	1.45 ± 0.30
	**PLA**	1.32 ± 0.14	1.27 ± 0.27	1.32 ± 0.14	1.24 ± 0.22	1.22 ± 0.07	1.23 ± 0.16
	**CON**	1.38 ± 0.13	1.32 ± 0.15	1.38 ± 0.15	1.26 ± 0.14	1.37 ± 0.12	1.25 ± 0.11
**Mean Power (W∙kg**^**-0.67**^**)**	**CR + SB**	1.12 ± 0.17	1.54 ± 0.17^a^	1.11 ± 0.13	1.51 ± 0.18^a^	1.05 ± 0.16	1.39 ± 0.20^a^
	**CR**	1.17 ± 0.14	1.40 ± 0.23^a^	1.06 ± 0.12	1.22 ± 0.13^a^	1.01 ± 0.13	1.11 ± 0.12
	**SB**	1.08 ± 0.13	1.34 ± 0.20^a^	1.08 ± 0.14	1.31 ± 0.20^a^	1.02 ± 0.19	1.27 ± 0.23^a^
	**PLA**	1.11 ± 0.15	1.10 ± 0.20	1.14 ± 0.12	1.07 ± 0.17	1.04 ± 0.09	1.01 ± 0.11
	**CON**	1.14 ± 0.11	1.13 ± 0.10	1.17 ± 0.12	1.08 ± 0.10	1.09 ± 0.09	1.07 ± 0.10
**Fatigue Index (W∙S**^**− 1**^**)**	**CR + SB**	21.42 ± 6.66	20.56 ± 9.13	25.88 ± 7.89	20.86 ± 19.8	31.37 ± 7.68	24.52 ± 9.49
	**CR**	25.13 ± 7.41	25.04 ± 12.7	27.68 ± 11.7	29.05 ± 15.5	28.29 ± 11.2	32.78 ± 6.68
	**SB**	26.22 ± 9.28	13.45 ± 13.3	24.86 ± 9.23	15.80 ± 10.1	26.38 ± 9.76	19.04 ± 11.4
	**PLA**	26.13 ± 5.76	24.32 ± 13.0	25.37 ± 7.22	26.63 ± 12.2	18.39 ± 5.37	23.30 ± 10.4
	**CON**	29.06 ± 9.41	25.45 ± 9.90	27.97 ± 12.3	24.75 ± 8.70	27.84 ± 8.69	24.34 ± 10.1
**Successful Kicks (number)**	**CR + SB**	59.00 ± 3.02	58.63 ± 2.45	56.75 ± 2.49	69.13 ± 3.31	68.38 ± 3.25	68.50 ± 2.51
	**CR**	61.13 ± 1.13	58.00 ± 1.85	56.63 ± 2.20	65.00 ± 2.56	62.25 ± 2.31	59.38 ± 1.68
	**SB**	59.63 ± 1.19	59.62 ± 2.50	57.88 ± 1.96	66.50 ± 1.07	65.75 ± 1.16	64.50 ± 2.20
	**PLA**	60.38 ± 1.77	61.25 ± 1.16	58.62 ± 1.92	60.00 ± 3.21	59.00 ± 2.39	57.50 ± 2.14
	**CON**	59.13 ± 1.89	60.13 ± 2.64	57.75 ± 1.75	58.75 ± 1.67	57.50 ± 2.00	57.25 ± 2.25

^a^ = significantly different than baseline

#### Mean power

**Fig. 2:**
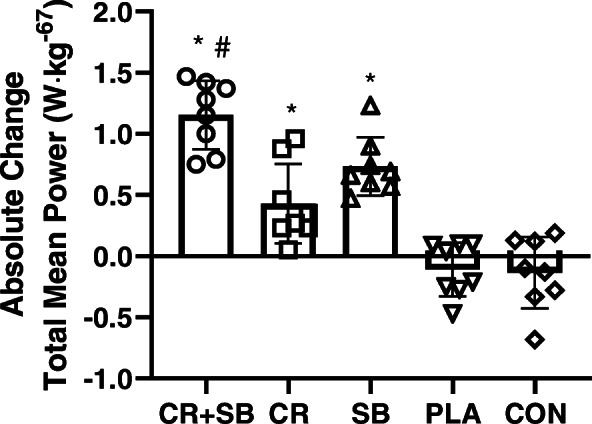
Mean and SD for absolute total relative MP change from baseline to post testing. Each dot represents an individual participant. * = significantly different than PLA and CON; # = significantly different than CR and SB

There was a significant bout x group x time interaction for MP (*P* < 0.001, ${\eta}_P^2$ = 0.600), as shown in Table 2. Post hoc analyses revealed a significant increase over time in bouts 1, 2, and 3 following CR + SB and SB (*P* < 0.001), whereas CR increased over time in bouts 1 (*p* = 0.03) and 2 (*p* = 0.02). There were no changes for PLA and CON over time (*P* > 0.05). There was a significant group x time interaction for total (all three bouts combined) mean PP (*P* < 0.001, ${\eta}_P^2$ = 0.789). Post hoc analysis revealed a significant increase over time for CR + SB (*P* < 0.001), CR (*P* = 0.003), SB (*P* < 0.001), with no changes in the PLA or CON groups. Absolute and percent change scores from baseline to post testing between groups was significant for total MP across the three bouts (Absolute: *P* < 0.001, ${\eta}_P^2$ = 0.789; Percent: *P* < 0.001, ${\eta}_P^2$ = 0.782), as shown in Fig. 2. Post hoc analysis revealed greater percent and absolute (Fig. 2) change scores for CR + SB (Percent Δ: 35.6 ± 9.6%) compared to CR (Percent Δ: 13.9 ± 11.0%; *P* < 0001), SB (Percent Δ: 22.9 ± 6.0%; *P* = 0.04), PLA (Percent Δ: − 3.6 ± 7.0%; *P* < 0.001) and CON (Percent Δ: − 3.6 ± 8.4%; *P* < 0.001). Changes following CR and SB were similar to each other (Absolute: *P* = 0.19, Percent: *P* = 0.24) and greater than PLA and CON (*P* < 0.001).

#### Successful kicks

There was a significant group x time interaction for total (sum of three bouts) successful kicks (*P* < 0.001, ${\eta}_P^2$ = 0.791), as shown in Table 2. Post hoc analysis revealed a significant increase over time (*P* < 0.001) for the CR + SB, CR, and SB groups with no changes in the PLA or CON groups.

Absolute and percent change scores from baseline to post testing between groups were significant for total successful kicks across the three bouts (Absolute: *P* < 0.001, ${\eta}_P^2$ = 0.791; Percent: *P* < 0.001, ${\eta}_P^2$ = 0.780). Post hoc analysis revealed greater change scores for CR + SB (Absolute Δ: 28.63 ± 6.59 n; Percent Δ: 16.46 ± 3.97%) compared to CR (Absolute Δ: 10.88 ± 8.61 n, *P* < 0.001; Percent Δ: 6.28 ± 5.11%; *P* < 0001), PLA (Absolute Δ: − 3.75 ± 6.61 n, *P* < 0.001; Percent Δ: − 2.04 ± 3.61%; *P* < 0.001) and CON (Absolute Δ: − 3.50 ± 8.07 n, *P* < 0.001; Percent Δ: − 1.87 ± 4.43%; *P* < 0.001), however, CR + SB was similar to SB (Absolute Δ: 19.63 ± 4.37 n, *P* = 0.098; Percent Δ: 11.12 ± 2.71%, *P* = 0.085). Changes following were similar between CR and SB which were both greater than PLA and CON.

### Blood lactate

**Fig. 3:**
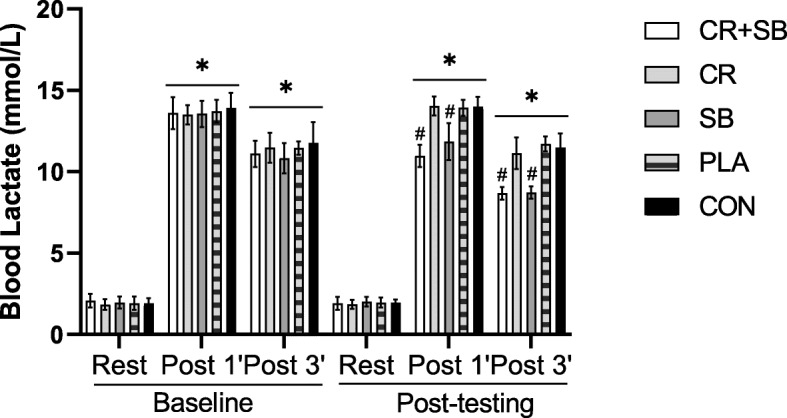
Mean and SD values of blood lactate in six times of measurements. * = significant increase from rest. # = significantly different than CR, PLA, and CON

There was significant group x time interaction for BL (*P* < 0.001, ${\eta}_P^2$ = 0.596), as shown in Fig. 3. At baseline, BL increased (*P* < 0.001) from resting to post-exercise in all groups with no differences at any time point (*P* > 0.05). The BL in CR + SB and SB were significantly lower immediately and 3 min after the test compared to CR, PLA, and CON (*P* < 0.001).

### Ratings of perceived exertion

There was a significant bout x group x time interaction for RPE (*P* < 0.001, ${\eta}_P^2$ = 0.0.339). Post hoc analysis revealed similar increases each bout during baseline testing between groups. During post-testing, CR + SB was significantly different from PLA following bouts 2 and 3 (*P* < 0.05). CR and SB alone were significantly different from PLA after bout 1 and PLA and CON after bout 2 (data not shown).

## Discussion

The main findings of the present study were that PP and MP increased following 5 days of supplementation with CR and SB, alone and in combination. In partial support of our hypothesis, there was a greater change in MP over time following co-ingestion of CR and SB compared to only CR and SB. However, co-ingestion of CR and SB did not enhance PP compared to CR and SB alone. Following SB supplementation (CR + SB and SB alone) post-exercise BL concentrations were attenuated compared to CR, PLA, and CON.

Mechanistically, acute and chronic ingestion of SB elevates blood HCO3- and enhances extracellular buffering capacity [19]. Theoretically, SB leads to a higher efflux of free hydrogen ions (H^+^) and lactate from skeletal muscle during intense physical exercise, thus postponing fatigue and enhancing high-intensity exercise performance [19, 32]. In the present study, SB enhanced exercise performance; however, contrary to previous studies reporting higher post exercise BL following SB in combat athletes [3, 9, 11] we found a lower post-exercise lactate concentration compared to that in PLA, CON, and CR groups. Given that BL concentrations represent the aggregate of anaerobic glycolytic production and lactate clearance. These findings may have been due to inter-individual heterogeneity in aerobic capacity possibly influencing lactate removal [33]. Therefore, future research with direct measures is warranted.

In line with previous literature, SB improved MP and PP compared to PLA and CON. For instance, Tobias et al. [11] and Oliveira et al. [34] found that 5–7 days of SB (500 mg∙kg-1) enhanced repeated Wingate performance by 7 and 2.9%, respectively. Ergogenic effects of chronic SB supplementation also were observed during repeated Anaerobic Sprint Test in elite soccer players [35]. Furthermore, a recent meta-analysis reported that chronic (500 mg∙kg^-1^ for 5 d) but not an acute bolus of SB improved PP and MP during anaerobic performance [19]. In support of our finding, a meta-analysis by Lopes-Silva and colleagues [19] reported higher PP after chronic SB supplementation. Although, the main energy system to generate higher PP largely depends on the PCr pathway, it has been well documented that the glycolytic pathway has already been activated to synthesize ATP within this time frame [6, 19]. Our findings provide further support that 5 days’ supplementation protocol was effective with no reported adverse effects which makes this strategy applicable in real sporting events.

With respect to combat sports, SB supplementation has been advantageous to enhance sport performance, including a karate-specific aerobic test (KSAT) [9], as well as a simulated matches of taekwondo [3], boxing [12], and a Judo [10], which supports our findings in taekwondo athletes during TAIKT .

The CR supplementation, which is known to enhance muscle CR and phosphocreatine (PCr) stores [25], improves anaerobic performance through several potential mechanisms [8]. Elevated PCr stores enhance the capacity to rephosphorylate ATP [36] and delay contributions from the glycolytic energy system. Importantly, PCr hydrolysis consumes a H^+^ ion and acts as a buffer against acidosis during exercise [6, 15]. CR also aids in the shuttling of high-energy phosphate metabolites between sites of ATP production (i.e. mitochondria) and the cytosol, leading to increased oxidative recovery [13]. In addition, CR increases calcium re-uptake into the sarcoplasmic reticulum augmenting myofibrillar cross-bridge cycling and force development [37]. It is estimated that the ATP-PCr energy system contributes 26–30% of total energy production during an attack and counter-attack action in taekwondo [1, 14], and therefore CR supplementation may enhance taekwondo performance. We provide evidence that CR supplementation for 5 days (sufficient to saturate the muscle) enhances MP and PP compared to PLA and CON. In agreement with our findings, short-term and long-term CR supplementation have been shown to improve six 35-m maximal sprints using a running anaerobic sprint test in male and female soccer players [38, 39].

Concerning combat sports, CR supplementation has shown equivocal findings. For example, CR failed to enhance anaerobic power in trained wrestlers [40] and taekwondo athletes [41], while CR supplementation in elite wrestlers improved Wingate performance [42]. Five days of CR (20 g∙day^− 1^) increased MP during a 30-s Wingate in elite wrestlers [42]. Differences between studies may be associated with methodological differences including training status, fiber type differences, baseline CR levels, and conducted anaerobic test. The present study was the first to examine CR on taekwondo athletes using sport-specific test and found positive results.

Although, CR and SB co-ingestion did not result in a higher PP compared to when CR and SB consumed separately, but it was higher than PLA and CON. These findings partially support previous research examining CR and SB co-ingestion [16, 17]. Barber et al. reported a 7% improvement following 2 days of CR and SB on relative peak power during repeated Wingate compared to CR (4%) and PLA [16]; however, this study did not include a SB alone condition for further comparison. In another study, CR co-ingestion over 6 days (20 g∙day^− 1^) and an acute dose of SB resulted in a 1.5% improvement in swimming performance in the second repeat of a 2 × 100-m freestyle swimming test compared to PLA [17]. On the other hand, Griffen et al. reported no additional ergogenic effect of 7 days of CR (20 g/day) and an acute 300 mg∙kg^− 1^ dose of SB supplementation compared to CR and SB alone on indices of mechanical power output during repeated Wingate test [18]. The mixed results could be attributed to some methodological differences existing between studies. For example, Barber et al. [16] and Mero et al. [17] did not include a SB alone condition thus making it difficult to interpret whether CR + SB was superior to either supplement alone. Another discrepancy of previous research was employing the acute SB supplementation strategy and/or the short (i.e., 2 days) CR loading period [19]. In the present study, a chronic SB (500 mg∙kg^− 1^ for 5 days) supplementation strategy was used with 5 days of CR loading which is known to saturate the muscle [25]. Although the co-ingestion did not further augment PP compared to either supplement alone, but MP was augmented to a greater degree following CR + SB compared to SB and CR alone. The precise mechanism(s) explaining the summative effect of CR + SB co-ingestion in the present study remains to be elucidated.

There are several limitations to the present study. First, we were unable to measure blood HCO3- and pH as well as intramuscular CR measures which would have provided a better mechanistic insight to explain observed differences in co-ingestion versus the single-ingredient conditions. Second, our participants were national or premier league caliber athletes. Due to training commitments, conducting a crossover study design (which would have enhanced the statistical power) was challenging to achieve. Third, we only assessed anaerobic performance by using a sport-specific test while future studies investigating other fitness components such as aerobic, muscular strength and power, flexibility, speed, and agility that are critical for taekwondo success are required [1, 28]. Lastly, we only included males with low participant sample size in each group. Future research in females with a larger sample size is required due to potential sex-based differences in response to creatine and sodium bicarbonate [8].

## Conclusion

Creatine and sodium bicarbonate supplementation have both been shown to enhance anaerobic performance in combat sports athletes. There is limited research using a sufficient CR loading phase with chronic SB supplementation on anaerobic performance. The current study found improvements in PP and MP compared to a PLA or CON when CR and SB were ingested alone using a taekwondo specific test. Co-ingestion of CR with SB further augmented MP compared to CR and SB alone. As such, coaches and athletes could consider utilizing these supplements to provide a possible performance advantage; however, individual responsiveness may need to be considered. Thus, short-term CR (20 g∙day^− 1^) and SB (500 mg∙kg^− 1^∙day^− 1^) alone enhance TAIKT performance compared to PLA and CON in trained taekwondo athletes. CR co-ingested with SB augments MP during an anaerobic sport-specific test compared to CR and SB alone.

## Data Availability

Data and publication materials are available from the corresponding author on reasonable request.

## Abbreviations

CR: Creatine
SB: Sodium bicarbonate
PLA: Placebo
CON: Control
TAIKT: Taekwondo Anaerobic Intermittent Kick Test
PP: Peak power
MP: Mean power
FI: Fatigue index
HHQ: Health history questionnaire
PAR-Q: Physical activity readiness questionnaire
RPE: Ratings of perceived exertion
LLM: Lower limb mass
KSAT: Karate-specific aerobic test
PCr: Phosphocreatine
